# Elevated postoperative IL-1β induces disorder of intestinal microenvironment and alteration of gut microbiota

**DOI:** 10.3389/fmicb.2026.1744636

**Published:** 2026-03-23

**Authors:** Wanyu Wang, Xinyang Liao, Jin Liu, Bin Liu, Cheng Zhou, Peng Liang

**Affiliations:** 1 Department of Anesthesiology, West China Hospital, Sichuan University, Chengdu, China; 2 State Key Laboratory of Oral Diseases, National Center for Stomatology, National Clinical Research Center for Oral Diseases, Department of Anesthesiology, West China Hospital of Stomatology, West China School of Stomatology, Sichuan University, Chengdu, China; 3 Department of Urology, Institute of Urology, West China Hospital, Sichuan University, Chengdu, China; 4 Laboratory of Anesthesia and Critical Care Medicine, National-Local Joint Engineering Research Centre of Translational Medicine of Anesthesiology, West China Hospital, Sichuan University, Chengdu, China; 5 Day Surgery Center, General Practice Medical Center, West China Hospital, Sichuan University, Chengdu, China

**Keywords:** gut micribiota, gut microenvironment, IL-1β, oxygen metablism, perioperative inflammation

## Abstract

**Introduction:**

Previous research hinted at the importance of postoperative gut dysbiosis prevention, but the mechanisms remained unclear, posing a challenge for prevention and therapy. This study aims to investigate the characteristics of postoperative dysbiosis and the underlying mechanisms.

**Methods:**

The clinical cohort investigated the perioperative change of gut microbiota in patients undergoing thoracoscopy and its relationship with peripheral inflammatory indicators. Gut microbiome was characterized by 16S rRNA gene sequencing and Bugbase phenotype analysis. In the laboratory study, a mouse model of surgery/anesthesia stress was established to further investigate the potentially underlying mechanisms.

**Results:**

Microbiome analysis revealed a decrease in alpha diversity and a shift from obligate anaerobes to aerobes/facultative anaerobes after thoracoscopy. Postoperative IL-1β was negatively correlated with obligate anaerobe abundance and positively correlated with facultative anaerobe abundance. Higher facultative anaerobe abundance was associated with increased risk of postoperative complications and longer hospital stays. In the mouse model, surgery and rIL-1β intervention mirrored the oxygen phenotype changes in clinical cohort, and the colonic epithelium exhibited decreased ATP levels and hypoxic staining scores, with increased lactic acid. Downregulating postoperative IL-1β with IL-1β siRNA mitigated colonic hypoxic environment impairment and gut microbiota oxygen phenotype changes induced by surgery/anesthesia stress.

**Conclusion:**

Postoperative gut dysbiosis involves a phenotypic shift of the gut microbiota from anaerobes toward aerobes and facultative anaerobes. This shift may be driven by an IL-1β–colonic epithelial oxygen metabolism–colonic oxygen environment–gut microbiota regulatory axis, offering potential insights for early risk stratification of severe postoperative complications and strategies to improve postoperative recovery.

## Highlights

This study observed a shift in postoperative gut microbiota from anaerobic to aerobic/facultative anaerobic, and indicated a potential regulatory axis of “IL-1β - colonic epithelium oxygen metabolism - colonic oxygen environment - gut microbiota,” providing insights for early identification and risk factor classification, for severe postoperative complications and improving recovery.

## Introduction

The human gut microbiome is a complex group of microorganisms in the intestine that plays a crucial role in maintaining various physiological functions (Backhed et al., 2005). Postoperative changes in gut microbiome can lead to complications like wound infections (Holder-Murray et al., 2021), neurocognitive disorders (Lu et al., 2022), sepsis, and organ failure, causing prolonged recovery and increased healthcare costs (Mustansir Dawoodbhoy et al., 2021; Schmitt et al., 2019; Zheng et al., 2017). However, the critical cause of postoperative gut dysbiosis is not fully understood, and understanding these mechanisms is essential for improving postoperative clinical outcomes.

The gut microbiome interacts with the immune system and is influenced by systemic inflammation, observed in various inflammatory diseases (Frank et al., 2007; Normann et al., 2013; Shimizu et al., 2006). Surgery/anesthesia stress induce systematic inflammation (Viswanathan and Dhabhar, 2005; Sheeran and Hall, 1997), characterized by increased inflammatory cytokines like Interleukin-1β (IL-1β) and Tumor Necrosis Factor-*α* (TNFα) (Viswanathan and Dhabhar, 2005; Sheeran and Hall, 1997; Ozcinar et al., 2014). Our previous research indicated that gut dysbiosis, initiated by antibiotics, disappeared in mice treated with cefazolin alone but persisted in mice with surgery and cefazolin 19 days after surgery, suggesting a link between postoperative inflammation and gut changes (Liang et al., 2018).

Studies have shown that, the functional characteristics of a microbial community are more representative and closely related to the environment than the taxonomic composition (Gibbons, 2017). For microbiome study, the most widely used 16S rRNA gene (16S) sequencing provides information about the overall composition but does not readily estimate the functional capability of individual microbiome (Morgan and Huttenhower, 2014). BugBase is an algorithm that predicts functional pathways and interpretable phenotypes of the gut microbiota (Ward et al., 2017), helping researchers understand the characteristics of microbiota changes and the clinical relevance.

Normally, over 90% of gut microbiota are obligate anaerobes, and the proportion of facultative anaerobes and aerobes, such as proteobacteria, is small (Byndloss and Baumler, 2018; Litvak et al., 2018). Previous research has found that the relative abundance of obligate anaerobes, including *Clostridium* and *Bifidobacterium*, significantly decreased postoperatively, while facultative anaerobes, such as *Enterobacter* and *Gamma-proteobacteria*, significantly increased in patients after gastrointestinal and cardiac surgery (Zheng et al., 2017; Wu et al., 2012; Aardema et al., 2019; Maekawa et al., 2020). These indicate a possible association between perioperative dysbiosis and changes in oxygen metabolism. However, it is unclear if such an alteration in oxygen metabolism is a general characteristic of gut dysbiosis after surgery, necessitating further studies involving different surgery types to map the postoperative gut microbiome landscape.

Most previous research on perioperative changes in gut microbiota focused on abdominal surgery. However, dysbiosis has been observed after non-abdominal surgeries, like cardiothoracic surgery (Zheng et al., 2017; Aardema et al., 2019; Maekawa et al., 2020) and orthopedic surgery (Jiang et al., 2019). Thoracoscopic surgery, a common surgery type avoiding direct mechanical stimulation and requiring fewer antibiotics, presents an opportunity to study the correlation between postoperative gut dysbiosis and systemic inflammation. Currently, there is no research on gut microbiome sequencing in patients undergoing thoracoscopic surgery.

This study aims to investigate the correlation between postoperative gut dysbiosis, systemic inflammation, and the clinical prognosis among patients undergoing thoracoscopic surgeries, and further to explore the characteristics of postoperative changes in the composition and oxygen metabolism phenotypes of gut microbiota and the underlying mechanisms.

## Results

### Patients characteristics

From June 2019 to January 2020, adult patients scheduled to receive thoracoscopic partial lung resection in a single surgical group were screened and all signed consent forms. Paired fecal samples before and after surgery were collected from 71 patients for final analysis. Included patients had an average age of 59.46 ± 11.01 years, with 37 males (52.11%) and 34 females (47.89%). For the Clavien-Dindo classification of postoperative complications, grade II complications included pneumonia (*n* = 17), while grade III and above complications included deep vein thrombosis (*n* = 2), unplanned intubation (*n* = 1), sepsis (*n* = 1), pulmonary embolism (*n* = 1), and upper gastrointestinal bleeding (*n* = 1) (Table 1).

**Table 1:** Basic characteristics of included patients.

Perioperative variables (*N* = 71)	Mean ± SD/Frequency (Percentage)
General information	
Age (year)	59.46 ± 11.01
Gender	
Male	37 (52.11%)
Female	34 (47.89%)
BMI, kg/m^2^	23.03 ± 2.63
Smoking history	
No	38 (53.52%)
Yes	33 (46.48%)
Drinking history	
No	39 (54.93%)
Yes	32 (45.07%)
Charlson comorbidity index	
0	16 (22.54%)
1	19 (26.76%)
2	15 (21.13%)
≥ 3	21 (29.58%)
Surgery information	
ASA grade	
Grade 2	58 (81.69%)
Grade 3	13 (18.31%)
Blood loss (mL)	52.46 ± 68.74
Fluid (mL)	685.21 ± 578.63
Anesthesia duration (min)	196.63 ± 80.44
Surgery duration (min)	133.25 ± 69.34
Postoperative information	
Postoperative antibiotics use	
No	51 (71.83%)
Yes	20 (28.17%)
First postoperative defecation (day)	3.90 ± 1.66
Postoperative pathological diagnosis	
Adenocarcinoma	43 (60.56%)
Non- adenocarcinoma	28 (39.44%)
Postoperative complication classification (Clavien-Dindo)	
Grade 0	30 (42.25%)
Grade I	18 (25.35%)
Grade II	17 (23.94%)
≥ III	6 (8.45%)
Postoperative hospital stay (day)	6.55 ± 4.95

### Gut microbiota got disordered and the oxygen metabolism phenotypes were associated with clinical prognosis after thoracoscopy

**Figure 1:**
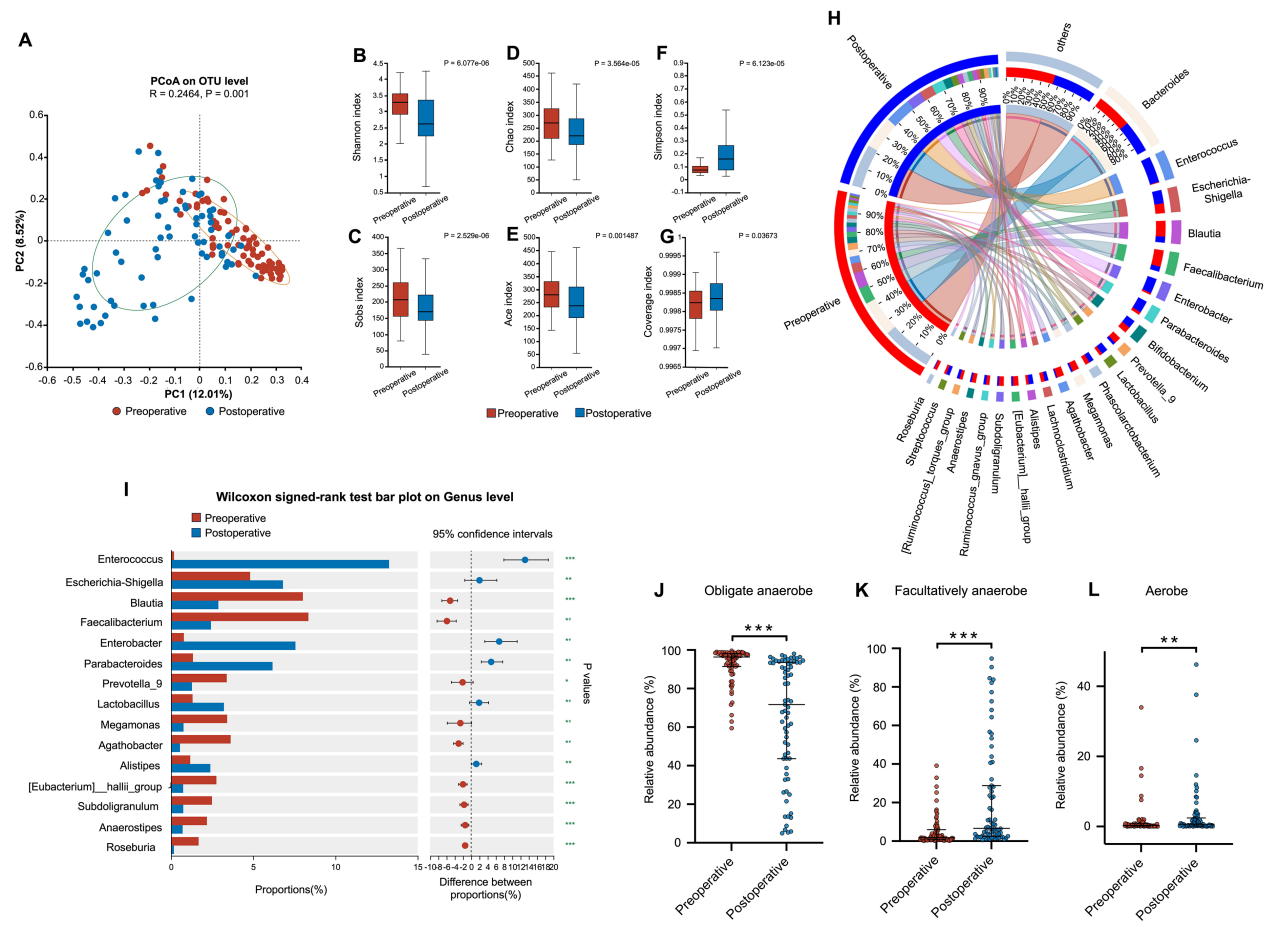
Postoperative change of gut microbiota in patients undergoing thoracoscopy. **(A)** Principal co-ordinates analysis (PCoA). PCoA plots based on Bray-Curtis distance metrics using the relative abundance of OTUs. PC1, Principal co-ordinate 1, explained 12.01% of the variation; PC2, Principal co-ordinate 2, explained 8.52% of the variation. **(B–G)** Alpha (*α*) diversity comparison of gut microbiota between the preoperative and postoperative samples according to the Shannon, Sobs, Chao, Ace, Simpson, and coverage indexes. The horizontal lines in the box plots represent median values; upper and lower ranges of the box represent the 75 and 25% quartiles. Statistical analysis was performed using Wilcoxon signed-rank test. **(H)** Perioperative changes of the microbiota composition at genus level (Circos). **(I)** Forest plot of the perioperative changes of the gut microbiota composition at genus level (Wilcoxon signed-rank test). **(J–L)** Comparison of the relative abundance of obligate anaerobe, facultative anaerobe and aerobe in perioperative gut microbiota based on Bugbase analysis (Wilcoxon signed-rank test). *: *p* < 0.05; **: *p* < 0.01; ***: *p* < 0.001.

Principal coordinate analysis (PCoA) shows a different clustering pattern of the fecal samples before and after thoracoscopic surgery, indicating a significant shift in the composition of gut microbiota (Figure 1A). The postoperative samples presented significantly lower Shannon, Sobs, Chao, and Ace indices compared to the preoperative samples, while the Simpson and Coverage indices are significantly higher than the preoperative samples (Wilcoxon rank sum test), suggesting a decrease in the alpha diversity of the gut microbiota in patients after thoracoscopic surgery (Figures 1B–G).

The composition of gut microbiota at genus level before and after the surgery are shown in Figure 1H,I. There was a significant decrease in the relative abundance of obligate anaerobes compared to preoperative level: Blautia (8.02% vs. 2.88%, *p* < 0.001), Faecalibacterium (8.36% vs. 2.43%, *p* < 0.001), Prevotella_9 (3.39% vs. 1.27%, *p* < 0.05), Megamonas (3.41% vs. 0.76%, *p* < 0.001), and Agathobacter (3.63% vs. 0.55%, *p* < 0.001). Meanwhile, the relative abundance of Enterococcus (0.18% vs. 13.26%, *p* < 0.001), Escherichia-Shigella (4.81% vs. 6.81%, *p* < 0.01), Enterobacter (0.78% vs. 7.57%, *p* < 0.01), Parabacteroides (1.33% vs. 6.17%, *p* < 0.001), Lactobacillus (1.31% vs. 3.22%, *p* < 0.001), and Alistipes (1.17% vs. 2.39%, *p* < 0.01) significantly increased postoperatively, most of which are facultative anaerobes or aerobes. The composition changes at the level of phylum and class are shown in Supplementary Figure 3.

The Bugbase phenotype prediction analysis revealed that the relative abundance of obligate anaerobe significantly decreased postoperatively (96.37% vs. 71.72%, *p* < 0.001), while the facultative anaerobe (1.79% vs. 6.63%, *p* < 0.001) and aerobe (0.29% vs. 0.65%, *p* < 0.01) significantly increased postoperatively (Figures 1J–L).

Meanwhile, we conducted regression models to explore the relationship between the oxygen metabolism phenotypes of gut microbiota and the postoperative prognosis. The linear regression analysis revealed that a higher relative abundance of postoperative facultative anaerobe was associated with a longer postoperative hospital stay, both in the unadjusted analysis [*β* = 0.94, 95% CI (0.50, 1.39), *p* < 0.001] and in the adjusted analysis [*β* = 0.67, 95% CI (0.19, 1.16), *p* = 0.009]. In the logistics regression analysis, a higher relative abundance of postoperative facultative anaerobe was found to be associated with a higher risk of clinically relevant postoperative complication (Clavien-Dindo Classification ≥ grade II), both in the unadjusted analysis [OR = 2.43, 95% CI (1.55, 3.79), *p* < 0.001] and in the adjusted analysis [OR = 4.33, 95% CI (1.40, 13.37), *p* = 0.011]. Besides, a higher relative abundance of postoperative obligate anaerobe was associated with a shorter postoperative hospital stay and a lower risk of clinically relevant postoperative complication in the unadjusted analysis, but the association was not significant in the adjusted analysis (Table 2).

**Table 2:** The associations between postoperative bacterial oxygen metabolism phenotypes and clinical outcomes.

Relative abundance of postoperative bacterial oxygen metabolism phenotypes	Postoperative hospital stay		Postoperative complications (Clavien-Dindo Classification ≥ grade II)	
	*β* (95% CI)	*p*	OR (95% CI)	*p*
Unadjusted model				
Facultative anaerobes (every 10%)	0.94 (0.50, 1.39)	<0.001	2.43 (1.55, 3.79)	<0.001
Obligate anaerobes (every 10%)	−0.55 (−0.91, −0.19)	0.004	0.77 (0.65, 0.92)	0.004
Aerobes (every 10%)	−0.27 (−1.77, 1.23)	0.724	1.05 (0.56, 1.97)	0.877
Adjusted model^1^				
Facultative anaerobes (every 10%)	0.67 (0.19, 1.16)	0.009	4.33 (1.40, 13.37)	0.011
Obligate anaerobes (every 10%)	−0.33 (−0.68, 0.03)	0.076	0.84 (0.64, 1.11)	0.214
Aerobes (every 10%)	−0.54 (−1.82, 0.74)	0.409	1.25 (0.48, 3.22)	0.648

^1^In the adjusted model, adjusting variables included age, gender, body mass index, drinking history, smoking history, Charlson comorbidity score, ASA grade, intraoperative blood loss, volume of administered fluid intraoperatively, anesthesia duration, operation duration, postoperative antibiotic prophylaxis, time to first bowel movement after surgery, postoperative pathological diagnosis, postoperative complications (Clavien-Dindo Classification).

### Inflammatory level increased postoperatively and postoperative IL-1β were associated with the oxygen metabolism phenotypes of gut microbiota

**Figure 2:**
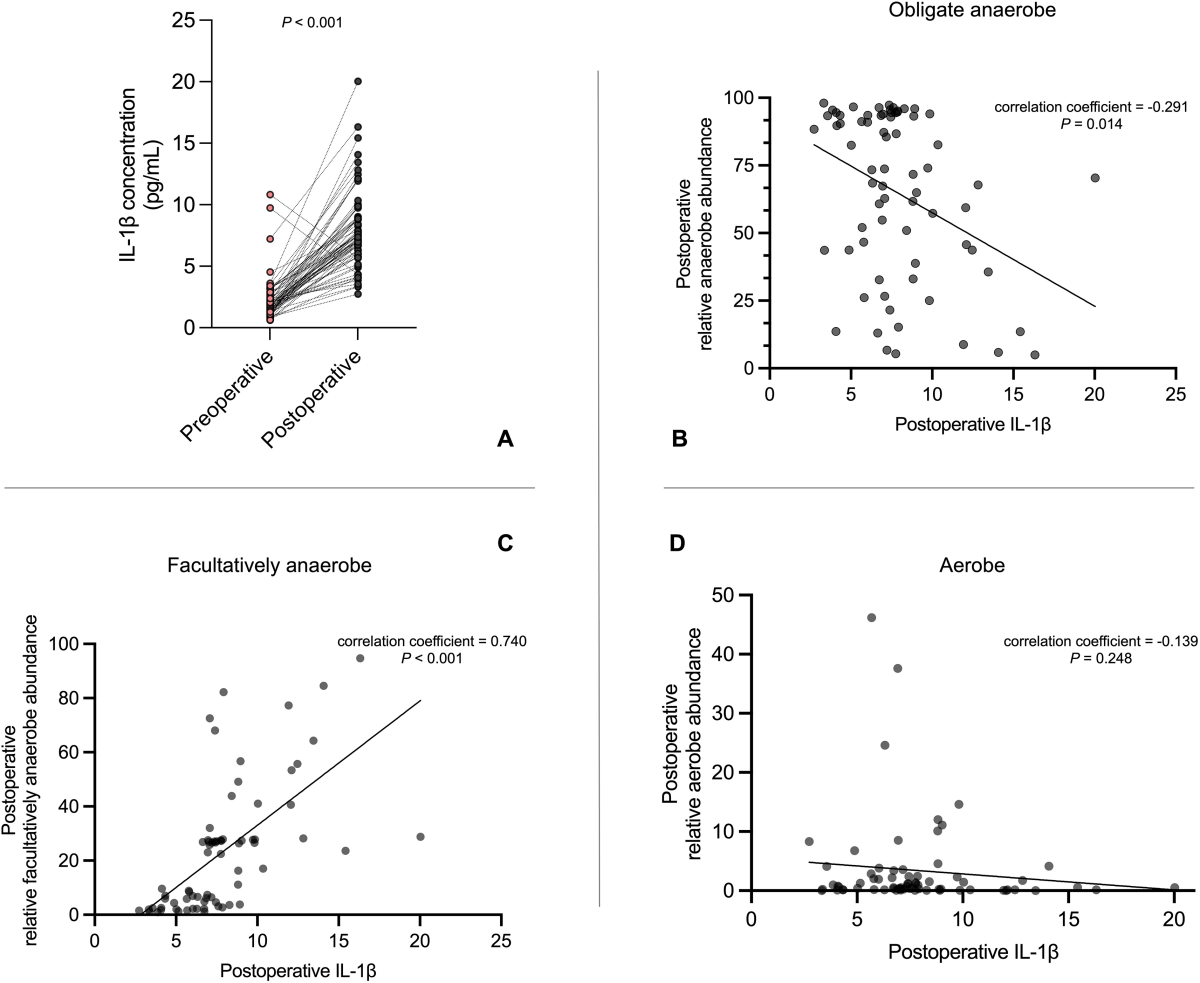
The association between postoperative IL-1β level and the oxygen metabolism phenotypes of postoperative gut microbiota. **(A)** Perioperative change of IL-1β level (paired Student’s *t*-test). **(B–D)** Linear correlation between postoperative IL-1β level and the relative abundance of obligate anaerobe, facultative anaerobe, and aerobe in postoperative gut microbiome.

After surgery/anesthesia stress, patients not only experienced the oxygen metabolism phenotype shift of gut microbiota, but also an elevation in systemic inflammation levels, with the level of IL-1β (Figure 2A), IL-6, CRP, PCT, and the neutrophil count all significantly increased postoperatively, while the lymphocyte count significantly decreased (Supplementary Table 1). Moreover, in the correlation analysis between postoperative inflammatory indicators and postoperative bacterial oxygen metabolism phenotypes (Supplementary Table 2), we found that the level of postoperative IL-1β was significantly negatively correlated with the relative abundance of postoperative obligate anaerobe (correlation coefficient −0.291, *p* = 0.014), and positively correlated with postoperative facultative anaerobe (correlation coefficient 0.740, *p* < 0.001; Figures 2B–D).

We further did multivariate linear regression analysis between postoperative IL-1β level and postoperative bacterial oxygen metabolism phenotypes. As a continuous variable, every 1 pg./mL increase of postoperative IL-1β resulted in 2.85% decrease of postoperative relative anaerobe abundance [95% CI (−5.35, −0.35); *p* = 0.030] and 3.41% increase of relative facultative anaerobe abundance [95% CI (1.84, 4.98); *p* < 0.001] in the model adjusted for all clinical confounding factors. As the current commonly recommended clinical cutoff value for IL-1β is 5 pg./mL, we also establish a regression model with IL-1β as a categorical variable. After adjusting for all clinical confounding factors, compared to patients with postoperative IL-1β < 5 pg./mL, patients with IL-1β ≥ 5 pg./mL had an 18.62% increase in the relative abundance of facultative anaerobic bacteria [95% CI (5.60, 31.63); *p* = 0.007] (Table 3).

**Table 3:** Postoperative linear regression analysis of IL-1β levels and postoperative microbiota oxygen metabolism phenotypes.

Variable	Relative abundance of postoperative Obligate anaerobes (%)		Relative abundance of postoperative Facultative anaerobes (%)		Relative abundance of postoperative Aerobes (%)	
	*β* (95% CI)	*p*	*β* (95% CI)	*p*	*β* (95% CI)	*p*
Unadjusted model						
Continuous IL-1β (pg/mL)	−3.45 (−5.57, −1.34)	**0.002**	4.59 (3.23, 5.95)	**<0.001**	−0.27 (−0.84,0.30)	0.359
Categorical IL-1β (clinical cutoff)						
<5 pg./mL	Ref		Ref		Ref	
≥5 pg./mL	−14.09 (−33.65, 5.47)	0.162	23.31 (9.11, 37.51)	**0.002**	1.65 (−3.38, 6.67)	0.524
Adjusted model^1^						
Continuous IL-1β (pg/mL)	−2.85 (−5.35, −0.35)	**0.030**	3.41 (1.84, 4.98)	**<0.001**	−0.17 (−0.91, 0.57)	0.654
Categorical IL-1β (clinical cutoff)						
<5 pg./mL	Ref		Ref		Ref	
≥5 pg./mL	−11.64 (−31.39, 8.12)	0.254	18.62 (5.60, 31.63)	**0.007**	1.53 (−4.12, 7.18)	0.598

^1^In the adjusted model, adjusting variables included age, gender, body mass index, drinking history, smoking history, Charlson comorbidity score, ASA grade, intraoperative blood loss, volume of administered fluid intraoperatively, anesthesia duration, operation duration, postoperative antibiotic prophylaxis, time to first bowel movement after surgery, postoperative pathological diagnosis, preoperative IL-1β levels, preoperative relative abundance of the certain oxygen phenotypes. Bold values indicate statistically significant differences (*p* < 0.05).

### Surgery/anesthesia stress induced elevation of systemic and intestinal IL-1β

**Figure 3:**
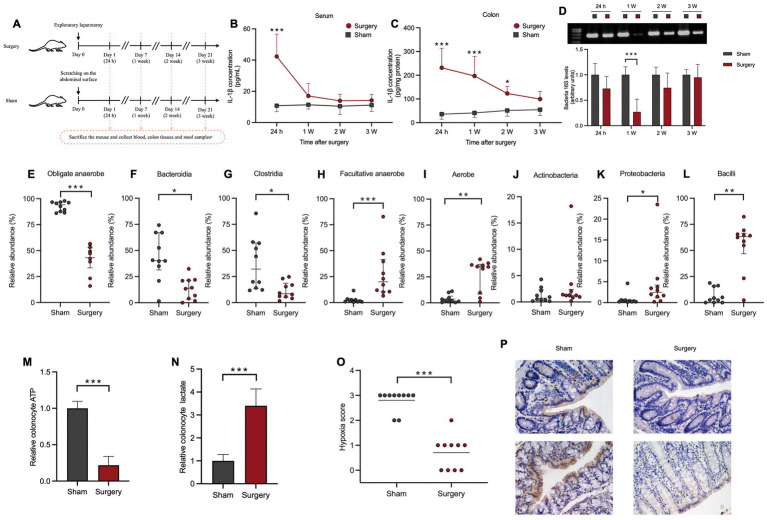
Surgery/anesthesia stress induced elevation of systemic IL-1β and the oxygen metabolism shift in gut microbiota as well as the colonic environment. **(A)** Experimental design. a, Mice were euthanized at 24 hours, 1 week, 2 weeks, and 3 weeks after the exploratory laparotomy, 10 mice/time point in each group. Serum, colon tissues, and fecal samples were collected for the measurement of IL-1β levels and detection of gut microbiota. **(B)** Change of IL-1β level in mice serum within 3 weeks after surgery. **(C)** Change of IL-1β level in mice colon within 3 weeks after surgery. **(D)** The 16S rRNA gene agarose gel electrophoresis of mouse gut microbiota within 3 weeks after surgery. **(E–G)** Comparison of relative abundance of the total obligate anaerobe and the main types of obligate anaerobes in mice gut microbiota 1 week after surgery between two groups. **(H–L)** Comparison of relative abundance of the total facultative anaerobe, total aerobe and the main types of facultative anaerobes/aerobes in mice gut microbiota 1 week after surgery between two groups. **(M,N)** Comparison of the relative expression of ATP and lactic acid in colon epithelial cells of mice between two groups. **(O)** Colonic epithelial hypoxia staining score of mice in two groups. (0, no hypoxia; 1, mild/focal hypoxia; 2, moderate/multifocal hypoxia; 3, severe/diffuse hypoxia) **(P)** Colonic epithelial hypoxia staining of mice in two groups. *: *p* < 0.05; **: *p* < 0.01; ***: *p* < 0.001.

To investigate the potential association between microbiota and inflammation across different surgical types, in the animal experiment, a surgery/anesthesia stress mice model was developed using exploratory laparotomy. Firstly, we measured the serum and colon IL-1β levels and assessed microbial quantity in mouse fecal samples using 16S agarose gel electrophoresis at multiple timepoints to track the trends in systemic IL-1β and gut microbiota changes following surgery/anesthesia stress (Figure 3A). In the surgery group, serum IL-1β surged at 24 h post-surgery (*p* < 0.001), gradually declining afterwards. By 1 week post-surgery, serum IL-1β in the surgery group did not significantly differ from the sham group (Figure 3B). Meanwhile, colon IL-1β levels in the surgery group were notably higher than the sham group at 24 h (*p* < 0.001), 1 week (*p* < 0.001), and 2 weeks (*p* < 0.05) post-surgery. However, by 3 weeks post-surgery, there was no significant difference in colon IL-1β levels between the surgery and sham group (Figure 3C).

### After surgery/anesthesia stress, the gut microbiota shifted from obligate anaerobic to aerobic and the hypoxic microenvironment in gut was destroyed

The 16S agarose gel electrophoresis results revealed that 24 h after surgery, the surgery group had a slightly lower bacterial quantity than the sham group (*p* = 0.11), which significantly decreased (*p* < 0.001) at 1-week post-surgery, and gradually returned to normal levels since then (Figure 3D).

Since the most severe gut microbiota disruption was observed at 1 week post-surgery, we conducted subsequent microbiota sequencing analysis using fecal samples collected at this time point. In the Bugbase prediction analysis of oxygen metabolism phenotypes, the relative abundance of obligate anaerobes in the surgery group (48.11%) was significantly lower than that in the sham group (94.08%) (*p* < 0.001). Specifically, the relative abundance of two major obligate anaerobes, Bacteroidetes (13.84% vs. 40.37%, *p* < 0.05) and Clostridia (8.50% vs. 32.13%, *p* < 0.05), was significantly lower in the surgery group (Figures 3E–G). Compared to the sham group, the relative abundance of facultative anaerobes (20.05% vs. 1.90%, *p* < 0.001) and aerobes (34.80% vs. 1.98%, *p* < 0.01) in the surgery group significantly increased. Bacilli, a class of firmicutes, along with actinobacteria and proteobacteria, were the main facultative anaerobes/aerobes in the gut microbiota. In the surgery group, the relative abundance of proteobacteria (2.47% vs. 0.40%, *p* < 0.05) and Bacilli (63.31% vs. 3.48%, *p* < 0.01) was significantly higher than in the sham group, while the relative abundance of actinobacteria showed no significant difference between two groups (Figures 3H–L).

Both clinical patients undergoing thoracoscopic surgery and mice subjected to abdominal exploration in animal experiments exhibited changes in the composition of gut microbiota postoperatively. While these changes were diverse, they all displayed a common shift in the overall metabolic pattern from anaerobic to aerobic. Hence, we hypothesized that such a dysbiosis after surgery/anesthesia stress may be associated with alterations in colonic oxygen environment. Therefore, we tried to measure the ATP and lactate levels within colonic cells and performed hypoxia staining on colonic epithelium. Figures 3M,N demonstrated that the surgery group exhibited reduced ATP (*p* < 0.001) and increased lactate level (*p* < 0.001) in colonic epithelial cells. Hypoxia staining showed weaker staining and lower hypoxia scores in the surgery group compared to sham group (*p* < 0.01) (Figures 3O,P). This suggested that under surgery/anesthesia stress, aerobic respiration weakened while anaerobic respiration strengthened in colonic epithelial, indicating a disrupted postoperative colonic hypoxic environment, aligning with the oxygen metabolism phenotype changes observed above.

### IL-1β plays a vital role in the surgery/anesthesia stress induced colonic oxygen environment disruption and gut microbiota dysbiosis

**Figure 4:**
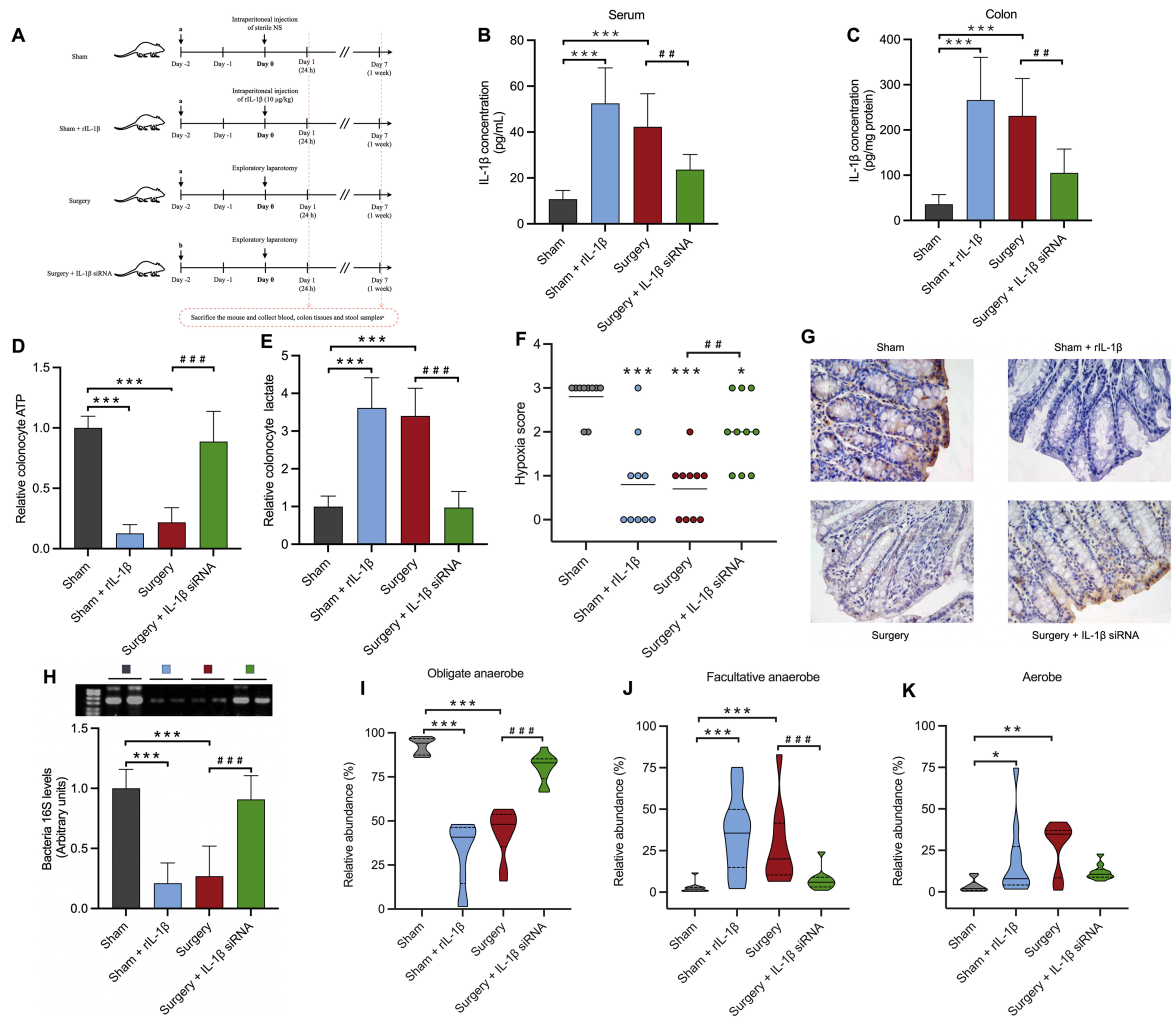
IL-1β mediates the colonic oxygen environment disruption and gut microbiota dysbiosis induced by surgery/anesthesia stress. **(A)** Experimental design. **(a)** Tail vein injection of Invivofectamine™ 3.0/chemically modified control siRNA complexes; **(b)** Tail vein injection of Invivofectamine™ 3.0/chemically modified IL-1β siRNA complexes; **(c)** Mice were euthanized at 24 h and 1 week after the exploratory laparotomy, 10 mice/time point in each group. Serum, colon tissues, and fecal samples were collected for the measurement of IL-1β levels and detection of gut microbiota. **(B,C)** Comparison of postoperative level of IL-1β in mice serum and colon 24 h after surgery in different groups. **(D,E)** Comparison of the relative expression of ATP and lactic acid in colon epithelial cells of mice 1 week after surgery in different groups. **(F)** Colonic epithelial hypoxia staining score of mice 1 week after surgery in different groups. (0, No hypoxia; 1, mild/focal hypoxia; 2, moderate/multifocal hypoxia; 3, severe/diffuse hypoxia) **(G)** Colonic epithelial hypoxia staining of mice 1 week after surgery in different groups. **(H)** The 16S rRNA gene agarose gel electrophoresis of mice gut microbiota 1 week after surgery in different groups. **(I–K)** Comparison of relative abundance of the total obligate anaerobe, total facultative anaerobe, and total aerobe in mice gut microbiota 1 week after surgery in different groups. *: *p* < 0.05; **: *p* < 0.01; ***: *p* < 0.001 vs. sham group. #: *p* < 0.05; ##: *p* < 0.01; ###: *p* < 0.001 vs. surgery group.

The clinical cohort has established a strong link between elevated postoperative IL-1β level and the oxygen metabolism phenotype of gut microbiota. Such an association was further validated in a mouse model. To investigate the role of IL-1β in the surgery/anesthesia stress induced colonic oxygen environment disruption and gut microbiota dysbiosis, we applied the chemically modified IL-1β siRNA and recombinant IL-1β (rIL-1β) to modulate IL-1β levels in the subsequent *in vivo* study (Figure 4A). After identifying the most effective siRNA sequence in cells, the sequence with the best *in vitro* knockdown efficiency was chemically synthesized, modified, and tested *in vivo*. The chemically modified IL-1β siRNA was administered via tail vein injection in mice, and its concentration in mouse plasma was quantified using HIT qRT-PCR (Landesman et al., 2010). The *in vivo* knockdown efficacy was further validated in a mouse LPS model (Supplementary Figure 2).

At 24 h post-surgery, the surgery + IL-1β siRNA group exhibited significantly lower IL-1β levels in serum (*p* < 0.01) and colon (*p* < 0.01) compared to surgery group. Conversely, the sham + rIL-1β group, 24 h after intraperitoneal rIL-1β injection, showed significantly higher IL-1β levels in serum (*p* < 0.001) and colon (*p* < 0.001) compared to sham group (Figures 4B,C).

Similar to surgery group, mice in the rIL-1β group displayed reduced ATP (*p* < 0.001), increased lactate (*p* < 0.001) and lower hypoxia staining scores (*p* < 0.001) in colonic epithelial tissues (Figures 4D–G), indicating that IL-1β could shift colonic epithelial cell toward enhanced anaerobic and reduced aerobic respiration, and cause a disruption to the normal colonic hypoxic environment. The 16S agarose gel electrophoresis showed a significant reduction in the total bacteria quantity 1 week after rIL-1β injection (*p* < 0.001) in the rIL-1β group (Figure 4H**)**. Bugbase phenotype prediction indicated a significant decrease in the relative abundance of obligate anaerobes (40.81% vs. 94.08%) (*p* < 0.001) and the increase in aerobes (7.91% vs. 1.98%, *p* < 0.001) and facultative anaerobes (35.60% vs. 1.90%, *p* < 0.05) in rIL-1β group compared to the sham group (Figures 4I–K). These findings indicated that under high IL-1β levels, gut microbiota dysbiosis occurred. The disruption of the hypoxic environment is unfavorable for obligate anaerobes but beneficial for aerobes and facultative anaerobes.

Mice in the surgery + IL-1β siRNA group exhibited recovered colonic hypoxic environment, with increased ATP (*p* < 0.001), reduced lactate (*p* < 0.001), and higher hypoxia staining scores close to normal level (*p* < 0.01). These results demonstrated that lowering IL-1β levels could correct the disruption of the colonic hypoxic environment caused by surgery/anesthesia stress (Figures 4D–G).

By reducing postoperative serum IL-1β levels with chemically modified IL-1β siRNA, the reduction in bacteria quantity caused by surgery/anesthesia stress were corrected (*p* < 0.001). Compared to the surgery group, the relative abundance of obligate anaerobes showed a restorative increase in the surgery + IL-1β siRNA group (*p* < 0.001). Furthermore, the relative abundance of facultative anaerobes and aerobes in the surgery + IL-1β siRNA group returned to sham levels, with a significantly lower relative abundance of facultative anaerobes compared to the surgery group (*p* < 0.01), while aerobes showed no significant difference (Figures 4H–K).

These results suggested that elevated IL-1β after surgery might be the primary cause of the shift in gut microbiota’s oxygen metabolism phenotype after surgery/anesthesia stress. Lowering IL-1β levels could alleviate the transition of the gut environment from low-oxygen to oxygen-rich, creating a more favorable environment for the survival of obligate anaerobes, thus maintaining gut microbiota homeostasis.

## Discussion

This study discovered that after thoracoscopic surgery, the gut microbiota shifted from anaerobic to aerobic/facultative anaerobic, which was associated with longer hospital stays and a higher risk of postoperative complications. Additionally, the postoperative peripheral IL-1β level was strongly related to the relative abundance of postoperative facultative anaerobes. We further confirmed such findings in a surgery/anesthesia stress mouse model. We discovered that, following surgery/anesthesia stress, elevated IL-1β was a key factor associated with reduced aerobic respiration in colonic cells, disrupting the hypoxic environment in the colon, and leading to dysbiosis in the gut microbiota characterized by a decrease in obligate anaerobes and an increase in facultative anaerobes/aerobes. Finally, we highlighted the feasibility and potential applications of preventing and treating perioperative dysbiosis by downregulating IL-1β levels, providing a conceptual framework for understanding and preventing the perioperative gut dysbiosis (Supplementary Figure 4).

The composition of gut microbiome is highly complex and exhibits substantial inter-individual variability, which can be influenced by many factors (Eckburg et al., 2005), such as diet (Turnbaugh et al., 2009), inflammatory bowel disease (Frank et al., 2007), diabetes (Qin et al., 2012), etc. Therefore, in the clinical cohort, to minimize the interference of non-study factors, we developed stringent exclusion criteria. Moreover, paired comparison was conducted to mitigate the influence of intra-individual variability.

While individual differences exist in gut microbiota composition, the functional characteristics of the gut microbiota tend to be more conserved across populations, geographic regions, and environments, and are closely linked to host health conditions (Human Microbiome Project Consortium, 2012; Levy et al., 2015). Previous perioperative studies have primarily focused on compositional changes in the gut microbiota, whereas perioperative functional or phenotypic alterations have received limited attention. Therefore, to better characterize shared perioperative microbiota features, we applied the Bugbase phenotype prediction analysis (Ward et al., 2017), in addition to taxonomic profiling.

Using Bugbase phenotype prediction, we found that a higher postoperative relative abundance of facultative anaerobes was associated with longer postoperative hospital stay and an increased risk of postoperative complications. Notably, most pathogenic bacteria fall into the category of facultative anaerobes. This observation is consistent with the World Health Organization’s list of antibiotic-resistant ‘priority pathogens,’ in which eight out of 12 listed pathogens are facultative anaerobes. Similar patterns have been reported in populations vulnerable to infectious diseases (André et al., 2021), providing a potential explanation for the increased susceptibility to infections and other complications following surgery.

Dysbiosis, although not precisely defined, is generally characterized by reductions in microbial diversity or substantial alterations in microbial composition (Shin et al., 2015; Rizzatti et al., 2017). Previous studies in both animal models and clinical patients have reported postoperative gut microbiota alterations following surgery/anesthesia stress (Wu et al., 2012; Jiang et al., 2019; Ohigashi et al., 2013; Howard et al., 2017; Ulker and Yildiran, 2019). In the present study, we observed distinct compositional changes in the gut microbiota following clinical thoracoscopic surgery in humans and exploratory laparotomy in mouse model. Although these two surgical procedures differ in approach and invasiveness, they were not intended to be procedurally equivalent. Importantly, Bugbase functional analysis revealed convergent changes in microbial oxygen metabolism phenotypes across these two distinct surgical models, characterized by a shift from obligate anaerobes toward facultative anaerobes and aerobes.

This categorization based on oxygen metabolism phenotype reveals similarities with previous studies across different surgical contexts. For example, investigations in cardiac surgery patients (Aardema et al., 2019; Maekawa et al., 2020) and elderly mice undergoing tibial fracture surgery (Jiang et al., 2019) reported a marked reduction in obligate anaerobic taxa, including Faecalibacterium, Anaerostipes, Roseburia, Alistipes, Ambiguous_axa, Lachnospiraceae_NK4A136, Lachnospiraceae_UCG, and Anaeroplasma, accompanied by an increase in facultative anaerobes such as Enterobacter, Bacteroides, and Staphylococcus.

Together, these consistent observations across diverse surgical settings suggest that alterations in microbial oxygen metabolism phenotypes may represent a common biological feature of surgery/anesthesia–associated gut dysbiosis rather than a procedure-specific effect. Nevertheless, further validation across additional surgical models and clinical populations is warranted.

Under physiological homeostasis, colonocytes consume the majority of available oxygen, thereby maintaining a hypoxic luminal environment that favors obligate anaerobes, which constitute more than 90% of the gut microbiome (Byndloss and Baumler, 2018; Litvak et al., 2018; Litvak et al., 2017; Byndloss et al., 2017). In inflammatory bowel disease, inflammation-associated impairment of epithelial oxygen metabolism has been proposed to disrupt intestinal hypoxia, leading to a shift from obligate anaerobes toward facultative anaerobes (Rigottier-Gois, 2013). Similar microbiota alterations have been reported in other inflammatory conditions, including necrotizing enterocolitis (Normann et al., 2013) and systemic inflammatory response syndrome (SIRS) (Shimizu et al., 2006). Based on these observations, we speculate that surgery/anesthesia–induced inflammatory stress may induce comparable alterations in the intestinal microenvironment, rendering it less favorable for obligate anaerobes and more permissive for facultative anaerobes and aerobes.

In the clinical cohort, postoperative IL-1β levels were significantly associated with the oxygen metabolism phenotype of the gut microbiota. In the mouse model, we further demonstrated that postoperative inflammatory responses correlated with microbiota oxygen phenotype changes and observed that IL-1β was sufficient to induce alterations in colonic epithelial oxygen metabolism, including reduced aerobic respiration and increased anaerobic respiration. As a consequence, reduced epithelial oxygen consumption may allow excess oxygen to diffuse into the intestinal lumen, thereby disrupting luminal hypoxia, as supported by decreased hypoxia staining scores in the colonic epithelium (Byndloss et al., 2017).

Although IL-6 exhibited a marked postoperative increase, our correlation analysis suggested that IL-1β, rather than IL-6, was more closely associated with changes in gut microbiota oxygen metabolism phenotype. In Supplementary Table 2, we systematically examined correlations between multiple postoperative inflammatory indicators—including IL-1β, IL-6, TNFα, CRP, PCT, lymphocyte count, monocyte count, and neutrophil count—and bacterial oxygen metabolism phenotypes.

Notably, postoperative IL-1β levels were significantly negatively correlated with the relative abundance of obligate anaerobes and strongly positively correlated with facultative anaerobes. In contrast, no significant correlations were observed between IL-6 levels and bacterial oxygen metabolism phenotypes. These findings suggest that, despite its pronounced elevation, IL-6 may not be directly linked to microbiota oxygen phenotype shifts in the perioperative context.

Together, these results support the hypothesis that IL-1β plays a more specific role in coupling postoperative inflammation to epithelial oxygen metabolism and gut microbial restructuring. Nevertheless, given the complex and interconnected nature of inflammatory signaling networks, we cannot exclude contributory or synergistic roles of other cytokines, including IL-6, which warrant further investigation.

Importantly, the temporal dynamics of IL-1β observed in our study suggest a phase-dependent contribution of distinct sources. We observed that circulating IL-1β levels increased early after surgery/anesthesia stress and declined at later time points, whereas IL-1β levels in colonic tissue remained elevated for a prolonged period. This pattern raises the possibility that early postoperative IL-1β elevation reflects a systemic inflammatory response to surgical and anesthetic stress, which may act as an initiating factor disrupting epithelial oxygen metabolism and gut microbial homeostasis.

Following this initial perturbation, gut microbiota dysbiosis itself may further exacerbate local intestinal inflammation, thereby promoting sustained IL-1β production within the colonic tissue. In this context, locally produced IL-1β, potentially derived from immune cells in the lamina propria, may reinforce epithelial metabolic dysfunction and perpetuate luminal oxygenation changes, forming a self-amplifying inflammatory–microbial feedback loop. Although the precise cellular sources of IL-1β at different stages were not directly identified in the present study, this two-phase model provides a plausible framework linking systemic inflammatory stress to persistent local intestinal inflammation and microbiota dysbiosis.

Notably, inflammatory cytokines such as IL-1β have been reported to induce mitochondrial dysfunction in various cell types, leading to impaired oxidative phosphorylation and altered cellular respiration (Yasuhara et al., 2005; Kowluru et al., 2011). In the present study, although direct mitochondrial functional assays were not performed, the observed reduction in epithelial aerobic respiration suggests that IL-1β–associated mitochondrial impairment may underlie the metabolic shift in colonic epithelial cells. Future studies incorporating direct assessments of mitochondrial function, such as membrane potential, reactive oxygen species production and respiratory chain activity, will be essential to fully delineate the downstream mechanisms by which IL-1β signaling remodels epithelial metabolism and the intestinal microenvironment.

In addition, hypoxia-inducible factor 1α (HIF-1α), a central regulator of cellular metabolism and inflammatory signaling, has been shown to interact with IL-1β–dependent pathways (Jung et al., 2003). Although HIF-1α was not directly assessed in the present study, the observed reduction in epithelial hypoxia staining following surgical stress suggests a disruption of the hypoxic niche that may involve HIF-1α–related pathways. Given the complex and potentially bidirectional regulation between IL-1β and HIF-1α, future studies employing *in vitro* epithelial models, such as colonoids or isolated intestinal epithelial cells, would be required to determine whether IL-1β directly modulates epithelial oxygen metabolism through HIF-1α–dependent mechanisms.

Covariates in the multivariable models were selected *a priori* based on clinical relevance and biological plausibility rather than data-driven procedures. Importantly, the associations remained directionally consistent across unadjusted and adjusted models (Table 2), as well as when IL-1β was analyzed as both a categorical and continuous variable (Table 3), supporting the robustness of the findings. Nevertheless, given the moderate sample size, residual overfitting cannot be entirely excluded. Thus, the results should be interpreted cautiously and considered hypothesis-generating pending validation in larger independent cohorts.

This study has several limitations. First, postoperative microbiota alterations were examined only in thoracoscopic surgery patients and in a laparotomy mouse model. Although findings from other surgical contexts support our conclusions (Aardema et al., 2019; Maekawa et al., 2020; Jiang et al., 2019), validation across additional surgical types is required to enhance generalizability. Second, only a single postoperative fecal sample was collected in the clinical cohort, precluding longitudinal assessment of dynamic microbiota changes. In addition, although the sex distribution was approximately balanced, the moderate sample size limited our ability to conduct sex-stratified analyses. Given that biological sex may influence inflammatory responses and gut microbiota composition, potential sex-specific effects warrant investigation in larger cohorts. Third, mechanistic exploration was limited. We did not perform cell-type–specific identification of IL-1β–producing immune populations, nor did we directly assess HIF-1*α* signaling or mitochondrial function in colonic epithelial cells. Therefore, the precise molecular intermediates linking IL-1β signaling and epithelial oxygen metabolism remain to be elucidated. Finally, this study was designed as an exploratory translational investigation, and formal a priori sample size calculation was not performed due to the lack of reliable effect size estimates in this emerging research area. Accordingly, our findings should be interpreted with caution and considered hypothesis-generating pending validation in larger, adequately powered studies.

In summary, our study identifies a reproducible shift in gut microbiota oxygen metabolism phenotype following surgery, characterized by a transition from obligate anaerobes to facultative anaerobes and aerobes, which is associated with adverse clinical outcomes. We further demonstrate that elevated IL-1β plays a key role in this process, supporting a potential regulatory axis of “IL-1β - colonic epithelial oxygen metabolism - intestinal oxygen microenvironment – gut microbiota.” These findings suggest that early monitoring of microbiota oxygen phenotype and inflammatory markers may aid in risk stratification for postoperative complications and provide a foundation for future interventions targeting inflammation and gut microenvironment to improve postoperative recovery.

## Materials and methods

### Clinical cohort and patient data

The study was approved by the Research Ethics Committee of West China Hospital of Sichuan University (project approval number 2019–846) and registered in Chinese Clinical Trial Registry (ChiCTR2200056824) on 2022/2/18. From June 2019 to January 2020, patients undergoing thoracoscopic partial lung resection in a single surgical group were screened and all signed consent forms. The inclusion and exclusion criteria are shown in Supplementary Figure 1. Paired peripheral blood as well as stool samples were collected before and after surgery. Fecal and serum samples were transported and stored in liquid nitrogen. Postoperative complications were graded using the Clavien-Dindo classification (Clavien et al., 2009). 16S rRNA gene sequencing was used to characterize the preoperative and postoperative gut microbiome and the online Bugbase phenotype prediction analysis was conducted.^1^ Enzyme-linked immunosorbent assay (ELISA) was used to quantify the perioperative concentration of Interleukin-1β (IL-1β) and Interleukin-6 (IL-6). The perioperative concentration of other inflammatory indicators including Tumor Necrosis Factor-α (TNF⍺), C-reactive protein (CRP), procalcitonin (PCT), lymphocyte count, monocyte count and neutrophil count were obtained by screening the hospital record (Supplementary Figure 1).

### General anesthesia and perioperative antibiotics

All patients underwent routine monitoring and general anesthesia on the day of surgery. For induction, patients were given injections of sulfentanyl (0.4 μg/kg), cisatracurium (0.2 mg/kg), and propofol (2 mg/kg). After induction, a left or right double-lumen endobronchial tube was inserted and the position of the endobronchial tube was confirmed using a fiberoptic bronchoscope. The anesthesia was maintained by remifentanyl (0.1 μg/kg/min) and 2% sevoflurane (0.8–1 MAC). Patients were subjected to either 6–8 mL/kg (two-lung ventilation) or 4–6 mL/kg (one-lung ventilation) according to the actual body weight, to maintain the SpO_2_ greater than 95% for two-lung ventilation and greater than 90% for one-lung ventilation. FiO_2_ was adjusted within the range of 0.5 to 0.8 for two-lung ventilation and maintained 1 for one-lung ventilation, at a respiratory rate of 12 to 15 /min. Intraoperative monitoring indicators included blood pressure, heart rate, SpO_2_, tidal volume, airway pressure and end-tidal carbon dioxide. The anesthesiologist adjusted the perioperative drugs and fluid plan according to the needs of the operation and the intraoperative parameters. After the operation, a regular patient-controlled analgesia pump was administered to control postoperative pain.

A single dose of prophylactic cefoxitin was given half an hour before surgery (2 g, ivgtt). And cefoxitin was administered 24-48 h after surgery (2 g, q8h, ivgtt) only when the surgeon thought postoperative antibiotic prophylaxis was necessary.

### Animal experimental groups

Two cohorts of animal experiments were applied (Figures 3A, 4A). Eight-week-old male mice were housed in pathogen-free conditions on a 12-h light/dark cycle with free access to water and food. All experiments were approved by the West China Hospital Ethics Committee and conducted in accordance with criteria outlined by the National Institutes of Health in the ‘The Guide for the Care and Use of Laboratory Animals’.

The first cohort aimed to monitor the dynamic changes of the IL-1β levels and the total bacterial quantity of the gut microbiota of mice following surgical/anesthesia stress, thus determining the timing for subsequent 16S sequencing of the microbiota and oxygen metabolism detection (Figure 3A). Mice in sham group underwent abdominal surface scratching with a cotton swab on Day 0. Mice in the surgery group underwent a laparotomy under 2.5–3% sevoflurane anesthesia on day 0, as described in our previous study (Liang et al., 2018), to establish the surgery/anesthesia stress model. Mice from both groups were sacrificed at 24 h (Day 1), 1 week (Day 7), 2 weeks (Day 14), and 3 weeks (Day 21) post-surgery, with 10 mice/time point in each group. Blood serum, colon tissues, and fecal specimens were collected at all timepoints.

In the second cohort, mice were randomly divided into 4 groups: sham group, sham + rIL-1β group, surgery group, surgery + IL-1β siRNA group (Figure 4A). The concentration of IL-1β in mice was downregulated by tail vein injection of chemically modified IL-1β siRNA (10 nmol) 2 days before surgery or upregulated by intraperitoneal injection of rIL-1β (10 μg/kg). Mice were euthanized at 24 h and 1 week after the anesthesia/surgery stress or the control condition, 10 mice/time point in each group. Serum, colon tissues, and fecal samples were collected at the each timepoint.

ELISA was used to measure IL-1β levels in both serum and colon tissues, while 16S agarose gel electrophoresis was employed to determine the bacterial quantity in mouse feces. The time point with the most severe gut microbiota disruption was determined according to the bacterial quantity result in the first cohort, and subsequent 16S sequencing and oxygen metabolism of gut microbiota (Bugbase phenotype prediction analysis) as well as colon tissues (ATP measurement /lactate measurement /hypoxia staining) at such a time point were conducted.

### Animal surgery/anesthesia stress model

The exploratory laparotomy mouse model of surgery/anesthesia stress was established. All mice were anesthetized by 2.5–3% sevoflurane and kept at spontaneous respiration with an inspiration of 50% O_2_. Rectal temperature was monitored and maintained at 37 °C with the aid of a heating blanket (366, Two Little Fishies, United States). The hair in the abdomen was shaved and the abdominal area was sterilized with iodine. Ropivacaine (0.1%, 3 mg/kg) was infiltrated to the incision site before surgery. The abdominal incision was made from the xiphoid process to the superior margin of symphysis pubis. The diaphragmatic surface of the liver, spleen, kidneys, and bladder were explored in sequence by a cotton tip wetted with sterile saline to mimic clinical exploratory laparotomy, after which, the peritoneum and skin were closed separately. All mice received a subcutaneous injection of ropivacaine (0.1%, 3 mg/kg) for postoperative analgesia. The total duration of inhalation anesthesia was 2 h for each mouse.

### IL-1β siRNA sequence selection

Mouse IL-1β siRNA were customized from Hippobio (Zhejiang, China). The sequences were as follows:

siRNA1: SS: GAUAGAAGUCAAGAGCAAAGU.
AS: UUUGCUCUUGACUUCUAUCUU.
siRNA2: SS: GGUUUGUCUUCAACAAGAUAG.
AS: AUCUUGUUGAAGACAAACCGU.
siRNA3: SS: AGAUAGAAGUCAAGAGCAAAG.
AS: UUGCUCUUGACUUCUAUCUUG.

The efficacy of each IL-1β siRNA was tested in an immortalized mouse macrophage cell line (Supplementary Figures 2A,B). Briefly, the cell line was cultured in specified primary cell culture medium (CM-M048, Procell, CN). Cell transfection was conducted using Lipofectamine™ 3,000 Reagent according to the manufacturer’s protocol (final concentration of siRNA in medium: 50 nM). After being transfected with either IL-1β siRNA or control siRNA for 48 h, cells were treated with LPS (final concentration of LPS in medium: 10 μg/mL) for 6 h and nigericin (final concentration of Nigericin in medium: 20 μM) for 30 min. Four hours after nigericin stimulation, collect the supernatant for IL-1β measurement (ELISA), and collect the cell lysate for real time quantitative PCR (RT-qPCR). Among three potential siRNAs targeting IL-1β, the siRNA with the best *in vitro* knockdown efficiency was selected and then chemically modified for the following *in vivo* study.

### Chemically modified IL-1β siRNA and the in vivo knockdown effect validation in a mouse LPS model

The IL-1β levels in the IL-1β siRNA group were downregulated by tail vein injection of chemically modified IL-1β siRNA (10 nmol) 2 days before surgery. Three customized IL-1β siRNA sequences were synthesized and tested in an immortalized mouse macrophage cell line to determine their effectiveness. The sequence with the best *in vitro* knockdown efficiency was then chemically synthesized and modified for the *in vivo* study.

The chemical modification of siRNA involved the terminal conjugation of cholesterol and the 2’-O-Methyl modification throughout the siRNA chain (Chernikov et al., 2019; Tai, 2019). The chemically modified siRNA was preserved under −80 °C and was resuspended in nuclease-free water before using. The resuspended siRNA was mixed with an equal amount of complexation buffer and then an equal amount of Invivofectamine™ 3.0 reagent. Incubate the mixture at 50 °C for 30 min, and then collect the mixture after short centrifugation, which can be used for tail vein injection.

To validate the *in vivo* knockdown effect of chemically modified siRNA, the chemically modified IL-1β siRNA or control siRNA was injected into the mouse tail vein (50 μM, 200 μL each mouse) on day 0. LPS was injected intraperitoneally on day 1, day 3, day 5, day 9 and day 14 after siRNA injection. The blood was collected 6 h after LPS injection. The concentration of chemically modified IL-1β siRNA in mouse plasma was detected by HIT qRT-PCR (heating-in-Triton quantitative reverse transcription polymerase chain reaction) as described below and the IL-1β level was detected using ELISA. The experimental process is shown in Supplementary Figure 5.

### HIT qRT-PCR for measurement of the siRNA concentration in mouse plasma

The HIT qRT-PCR (Landesman et al., 2010) method was used to quantify the concentration of siRNA in mice plasma.

#### Preparation of the standard curve and samples

(1) 50 μL mouse plasma from control mice (PBS treated) or siRNA-injected mice and 450 μL 0.25% Triton X-100 (diluted in PBS) were mixed in Eppendorf tubes and heat the mixture on a 95 °C heat block. Seven tubes of diluted mouse plasma from control mice were used for the standard curve preparation; (2) 50 μL siRNA (100 μM) was added into the 1st tube. Then the mixture in the 1st tube was vortexed; (3) 50 μL sample from the 1st tube was added to the 2nd tube and then the mixture in the 2nd tube was vortexed; (4) A 1:10 dilution was performed repeatedly until the 6th tube. No siRNA was added to the 7th tube of mouse plasma; (5) All tubes of diluted mouse plasma from control mice or siRNA-injected mice were heated on a 95 °C heat block for 10 min and cooled on ice for 10 min; (6) All tubes were centrifuged for 20 min at 20,000 g at 4 °C. Supernatant from all tubes was collected, put into new Eppendorf tubes, and stored on ice.

#### Reverse transcription

The reverse transcription reaction mixture was prepared as Supplementary Table 3, vortexed mildly and then cooled at 4 °C in a thermal cycler; (2) All supernatant from the last step was heated on a 95 °C heat block. 5 μL of supernatant from each tube in the last step was added to the reverse transcription reaction mixture. The reverse transcription reaction process was as follows: 16 °C, 30 min; 42 °C, 30 min; 85 °C, 5 min; 4 °C. The reverse transcription stem-loop primer was designed as follows: GTCGTATCCAGTGCGTGTCGTGGAGTCGGCAATTGCACTGGATACGAC.

#### PCR

The PCR mixture was prepared as Supplementary Table 4, vortexed in the dark. The mixture was then put into the real-time PCR machine (CFX96), and the PCR reaction process was as follows: 50 °C, 2 min; 95 °C, 10 min; 40 cycles of 95 °C for 15 s and 60 °C for 60 s. The PCR amplification primer was designed as follows:

forward primer: gggggTTGCTCTTGACTTCTA.

reverse primer: CAGTGCGTGTCGTGGAGT.

### Hypoxic staining of colon epithelia

Mice were treated with 60 mg/kg of pimonidazole HCl i.p. (HypoxyprobeTM-1 kit, Hypoxyprobe) 1 h prior to euthanasia. Euthanasia was performed by overdose of sodium pentobarbital (150 mg/kg, i.p.). Death was confirmed by cessation of respiration and lack of reflex response, and colon samples were immediately collected for further analysis. Colon samples were fixed in 10% buffered formalin and paraffin-embedded tissue was probed with mouse anti-pimonidazole monoclonal IgG1 and then stained with HRP conjugated goat anti-rabbit antibody (Jackon Immuno Research Laboratories). Hypoxic signals were then detected by DAB. Samples were scored based on the degree of colonic epithelial hypoxia (0: no hypoxia; 1: mild focal hypoxia; 2: moderate multifocal hypoxia; 3: intense diffuse hypoxia). Representative images were captured using a Zeiss microscope.

### ATP and lactate measurements of colon tissue

ATP and lactate measurement in colonocyte lysates was performed by using an ATP Colorimetry Assay Kit (Biovision) and a Lactate Colorimetry Assay Kit II (Biovision, Milpitas, CA) according to the manufacturer’s instructions.

### Real-time PCR

PCR was performed using Quantitect SYBR Green PCR Kit (Qiagen) according to the manufacturer’s protocol. Florescence was detected using a CFX96 system (BioRad). The profile was as follows: 95 °C for 3 min and 40 cycles of 95 °C for 10 s, 55 °C for 30 s. The primers were:

Mouse IL-1β (forward primer 5’-GAAATGCCACCTTTTGACAGTG-3’, reverse primer 5’-TGGATGCTCTCATCAGGACAG-3’),

Mouse beta actin (forward primer 5’- GGCTGTATTCCCCTCCATCG-3’, reverse primer 5’-CCAGTTGGTAACAATGCCATGT-3’),

Primer for siRNA validation in HIT qRT-PCR methods (forward primer 5’-GGGGGTTGCTCTTGACTTCTA-3’, reverse primer 5’-CAGTGCGTGTCGTGGAGT-3’).

### Enzyme-linked immunosorbent assays

Blood samples were centrifuged at 3,500 rpm for 20 min at 4 °C and the supernatant was collected for further ELISA experiments. Tissue samples were homogenized using an electric homogenizer and then lysed in RIPA. ELISA was performed using ELISA Kits (Human IL-1β ELISA kit, Sigma RAB0273; human IL-6 ELISA kit, Sigma RAB0306; Mouse IL-1β ELISA kit, ExCell Bio EM001) according to the manufacturer’s protocol. Florescence was detected on a Microplate Absorbance Reader at a 450 nm wavelength.

### Microbiota analysis

#### DNA extraction and PCR amplification

Microbial community genomic DNA was extracted from mouse stool samples using a E. Z. N. A.® soil DNA Kit (Omega Bio-tek, Norcross, GA, United States) according to the manufacturer’s instructions. Briefly, the DNA extract was checked on 1% agarose gel, and the DNA concentration and purity determined using a NanoDrop 2000 UV–vis spectrophotometer (Thermo Scientific, Wilmington, USA). The hypervariable region V3-V4 of the bacterial 16S rRNA gene were amplified with primer pairs 338F (5′-ACTCCTACGGGAGGCAGCAG-3′) and 806R (5′-GGACTACHVGGGTWTCTAAT-3′) by an ABI GeneAmp® 9,700 PCR thermocycler (ABI, CA, USA). The PCR amplification of the 16S rRNA gene was performed as follows: initial denaturation at 95 °C for 3 min, followed by 27 cycles of denaturing at 95 °C for 30 s, annealing at 55 °C for 30 s and extension at 72 °C for 45 s, and a single extension at 72 °C for 10 min, and the endpoint at 4 °C. The PCR mixtures contained 5 × TransStart FastPfu buffer 4 μL, 2.5 mM dNTPs 2 μL, forward primer (5 μM) 0.8 μL, reverse primer (5 μM) 0.8 μL, TransStart FastPfu DNA Polymerase 0.4 μL, template DNA 10 ng, and finally ddH_2_O up to 20 μL. PCR reactions were performed in triplicate. The PCR product was extracted from 2% agarose gel and purified using an AxyPrep DNA Gel Extraction Kit (Axygen Biosciences, Union City, CA, USA) according to the manufacturer’s instructions and quantified using a Quantus™ Fluorometer (Promega, USA).

#### Illumina MiSeq sequencing

Purified amplicons were pooled in equimolar portions and paired-end sequenced (2 × 300) on an Illumina MiSeq platform (Illumina, San Diego, United States) according to the standard protocols of Majorbio Bio-Pharm Technology Co. Ltd. (Shanghai, China). The raw 16S rRNA gene sequencing reads were demultiplexed, quality filtered by Fastp and merged by FLASH with the following criteria: (i) the 300 bp reads were truncated at any site receiving an average quality score of < 20 over a 50 bp sliding window, and truncated reads < 50 bp were discarded, as were reads containing ambiguous characters; (ii) only overlapping sequences longer than 10 bp were assembled according to their overlapped sequence. The maximum mismatch ratio of the overlap region was 0.2 and reads that could not be assembled were discarded; (iii) samples were distinguished according to the barcode and primers, and the sequence direction was adjusted, with exact barcode matching and 2 nucleotide mismatches in primer matching.

Operational taxonomic units with a 97% similarity cutoff were clustered using UPARSE^2^ and chimeric sequences were identified and removed. The taxonomy of each operational taxonomic unit representative sequence was analyzed by RDP Classifier^3^ against the 16S rRNA database (Silva SSU128) using a confidence threshold of 0.7. Fold changes of ratios (bacterial competitive index, mRNA levels and protein levels), percentages (flow cytometry) and bacterial numbers were transformed logarithmically prior to statistical analysis.

#### Bioinformatics analysis

Microbiome composition and diversity analysis: The Rank-Abundance curve and the Pan/Core species analysis curve were used to evaluate the adequacy of the clinical sample size and data volume for sequencing. The Alpha diversity index was used to analyze the colony richness and diversity of the intestinal flora before and after surgery. The Beta diversity index was used to analyze the similarities and differences of the intestinal flora before and after surgery. The composition and relative abundance of the intestinal flora at various taxonomic levels such as phyla and genus before and after surgery were displayed using colony histograms, Circos diagrams, etc. The analysis mentioned above was carried out on the online platform of Shanghai Meiji Biopharmaceutical Company.^4^

Bugbase phenotype prediction analysis: Based on the Greengene database, by integrating the gene information of the following three databases: IMG, KEGG and PATRIC, all the bacteria can be classified into 7 main phenotypes according to their OTU characteristics: Gram-positive, Gram-negative, Biofilm Forming, Pathogenic, Mobile Element Containing, Oxygen Utilizing (including aerobic, obligate anaerobic, facultatively anaerobic) and Oxidative Stress Tolerant. This part of the analysis was performed on the Bugbase public analysis platform.^5^

### Statistical analysis

Data analysis and statistical plotting were performed using GraphPad Prism ver. 8.0.0 (131) and R ver. 3.6.1. Continuous variables with a normal distribution are presented as mean ± standard deviation, otherwise as median and interquartile ranges. Categorical variables are presented as frequency (percentage). For comparisons of two groups of normally distributed continuous variables, when the homogeneity of variance was satisfied, a paired or independent sample t-test was employed, otherwise the Wilcoxon signed-rank test or t’-test was employed. For non-normally distributed continuous variables or rank variables, the Wilcoxon rank sum test was used. For three or more groups of normally distributed continuous variables, one-way analysis of variance (ANOVA) was used; otherwise, the Kruskal-Wallis rank-sum test was employed. Statistical significance was defined as a two-tailed *p*-value < 0.05.

## Data Availability

The raw data supporting the conclusions of this article will be made available by the authors, without undue reservation.

## Footnotes

1
https://bugbase.cs.umn.edu/

2 ver. 7.1, http://drive5.com/uparse/

3
http://rdp.cme.msu.edu/

4
https://cloud.majorbio.com/

5
https://bugbase.cs.umn.edu/
