# Supplementary material for: Elevated postoperative IL-1β induces disorder of intestinal microenvironment and alteration of gut microbiota
Source: Front Microbiol. 2026 Mar 23;17:1744636. doi: 10.3389/fmicb.2026.1744636 (PMC13050866; doi:10.3389/fmicb.2026.1744636)
Supplement: Supplementary file 1 [file Data_Sheet_1.docx]

**Supplementary Tables:**

**Supplementary Table 1:** The perioperative change of the peripheral blood inflammation indicators

| **Peripheral blood Inflammation indicators** | **Preoperative**  **(Mean ± SD)** | **Postoperative**  **(Mean ± SD)** | **P-values**  **(Pre vs. Post)** |
| --- | --- | --- | --- |
| IL-1β (pg/mL) | 2.26 ± 1.74 | 7.87 ± 3.19 | **<0.001** |
| IL-6 (pg/mL) | 4.35 ± 10.60 | 68.33 ± 74.47 | **<0.001** |
| TNF⍺ (pg/mL) | 6.61 ± 1.85 | 6.17 ± 2.02 | **0.040** |
| CRP (mg/L) | 5.02 ± 12.07 | 60.93 ± 40.62 | **<0.001** |
| PCT (ng/mL) | 0.05 ± 0.05 | 0.81 ± 1.24 | **0.001** |
| Lymphocyte count (10^9^/L) | 1.71 ± 0.53 | 1.03 ± 0.43 | **<0.001** |
| Monocyte count (10^9^/L) | 0.43 ± 0.16 | 2.08 ± 11.15 | 0.217 |
| Neutrophil count (10^9^/L) | 3.65 ± 1.43 | 10.79 ± 4.22 | **<0.001** |

**Supplementary Table 2:** Correlation analysis between postoperative inflammation indicators and postoperative bacterial oxygen metabolism phenotypes

| **Postoperative Inflammation Indicators** | **Relative Abundance of Postoperative  Obligate anaerobes (%)** | |  | **Relative Abundance of Postoperative  Facultative anaerobes (%)** | |  | **Relative Abundance of Postoperative  Aerobes (%)** | |
| --- | --- | --- | --- | --- | --- | --- | --- | --- |
|  | **Correlation coefficient** | **P-value** |  | **Correlation coefficient** | **P-value** |  | **Correlation coefficient** | **P-value** |
| IL-1β (pg/mL) | −0.291 | **0.014^*^** |  | 0.740 | **<0.001*** |  | −0.139 | 0.248 |
| IL-6 (pg/mL) | 0.044 | 0.716 |  | 0.145 | 0.229 |  | −0.103 | 0.392 |
| TNF⍺ (pg/mL) | 0.125 | 0.298 |  | −0.104 | 0.386 |  | −0.102 | 0.396 |
| CRP (mg/L) | 0.029 | 0.808 |  | 0.048 | 0.691 |  | 0.208 | 0.082 |
| PCT (ng/mL) | −0.050 | 0.680 |  | −0.027 | 0.824 |  | 0.072 | 0.551 |
| Lymphocyte count (10^9^/L) | 0.153 | 0.201 |  | 0.068 | 0.571 |  | 0.041 | 0.735 |
| Monocyte count (109/L) | 0.152 | 0.205 |  | 0.112 | 0.354 |  | −0.060 | 0.619 |
| Neutrophil count (109/L) | 0.303 | **0.010^*^** |  | -0.037 | 0.762 |  | −0.157 | 0.192 |

**Supplementary Table 3:** HIT qRT-PCR reverse transcription reaction mixture

| **Agents** | **Volume/tube** |
| --- | --- |
| 100mM dNTPs (with dTTP) | 0.15 μL |
| MultiScribeTM Reverse Transcriptase, 50 U/μL | 1.00 μL |
| 10✕ Reverse Transcription Buffer | 1.50 μL |
| RNase Inhibitor, 20 U/μL | 0.19 μL |
| 5✕ RT Primer | 3 µL |
| Nuclease-free Water | 4.16 μL |
| **Total** | **10 μL** |

**Supplementary Table 4:** HIT qRT-PCR mixture

| **Agents** | **Volume/tube** |
| --- | --- |
| TaqManTM Small RNA Assay 20X（0.2 μM TaqMan probe, 1.5 μM forward primer, 1.5μM reverse primer） | 1.00 μL |
| TaqMan 2 × Universal PCR Master Mix, No AmpErase UNG | 10.00 μL |
| Nuclease-free water | 7 μL |
| cDNA | 2 μL |
| **Total** | 20 μL |
