# Supplementary material for: Elevated postoperative IL-1β induces disorder of intestinal microenvironment and alteration of gut microbiota
Source: Front Microbiol. 2026 Mar 23;17:1744636. doi: 10.3389/fmicb.2026.1744636 (PMC13050866; doi:10.3389/fmicb.2026.1744636)
Supplement: Supplementary file 2 [file Data_Sheet_2.docx]

**Supplementary Figures:**

**
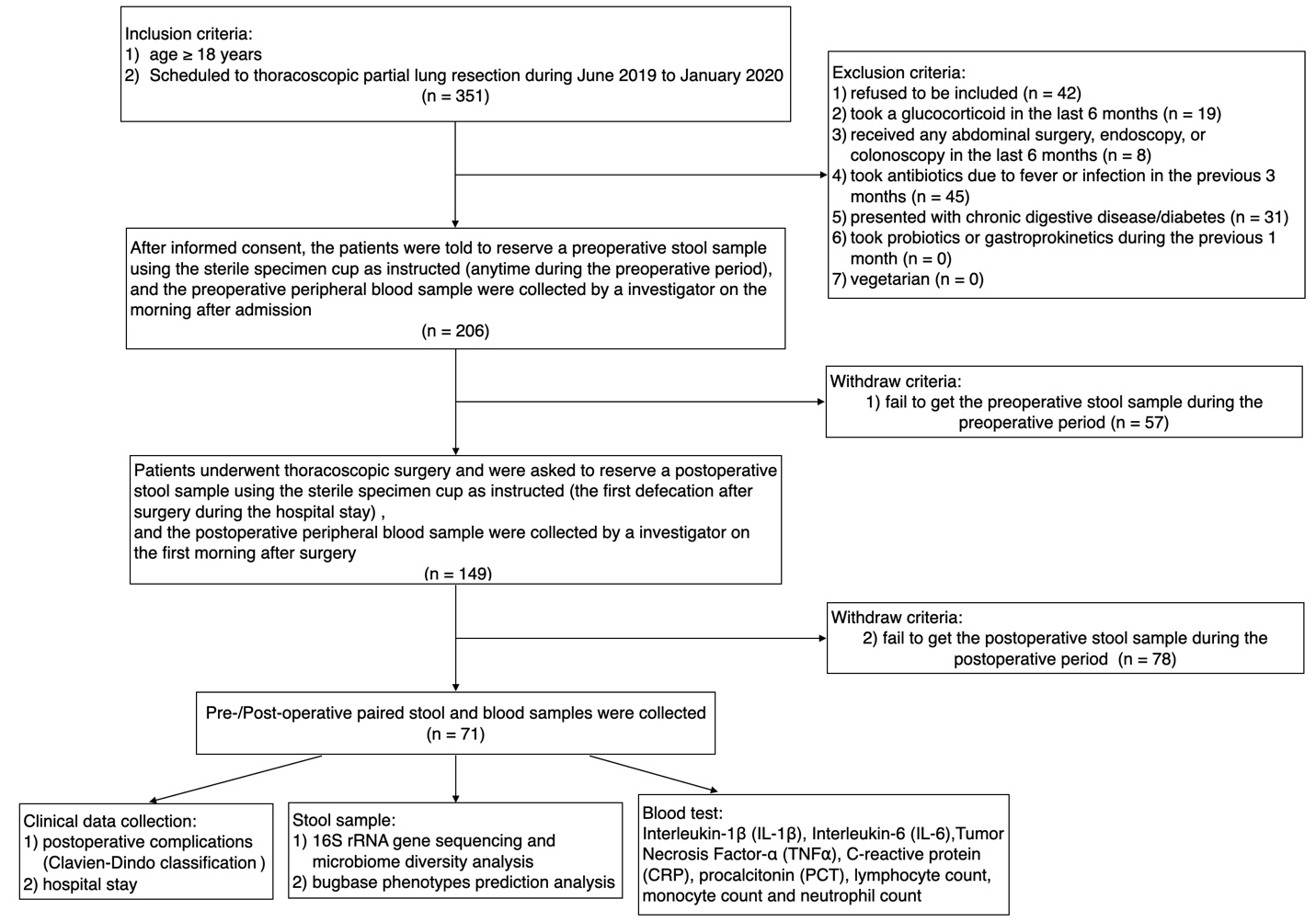
**

**Supplementary Figure 1:** Participant selection flowchart.

**
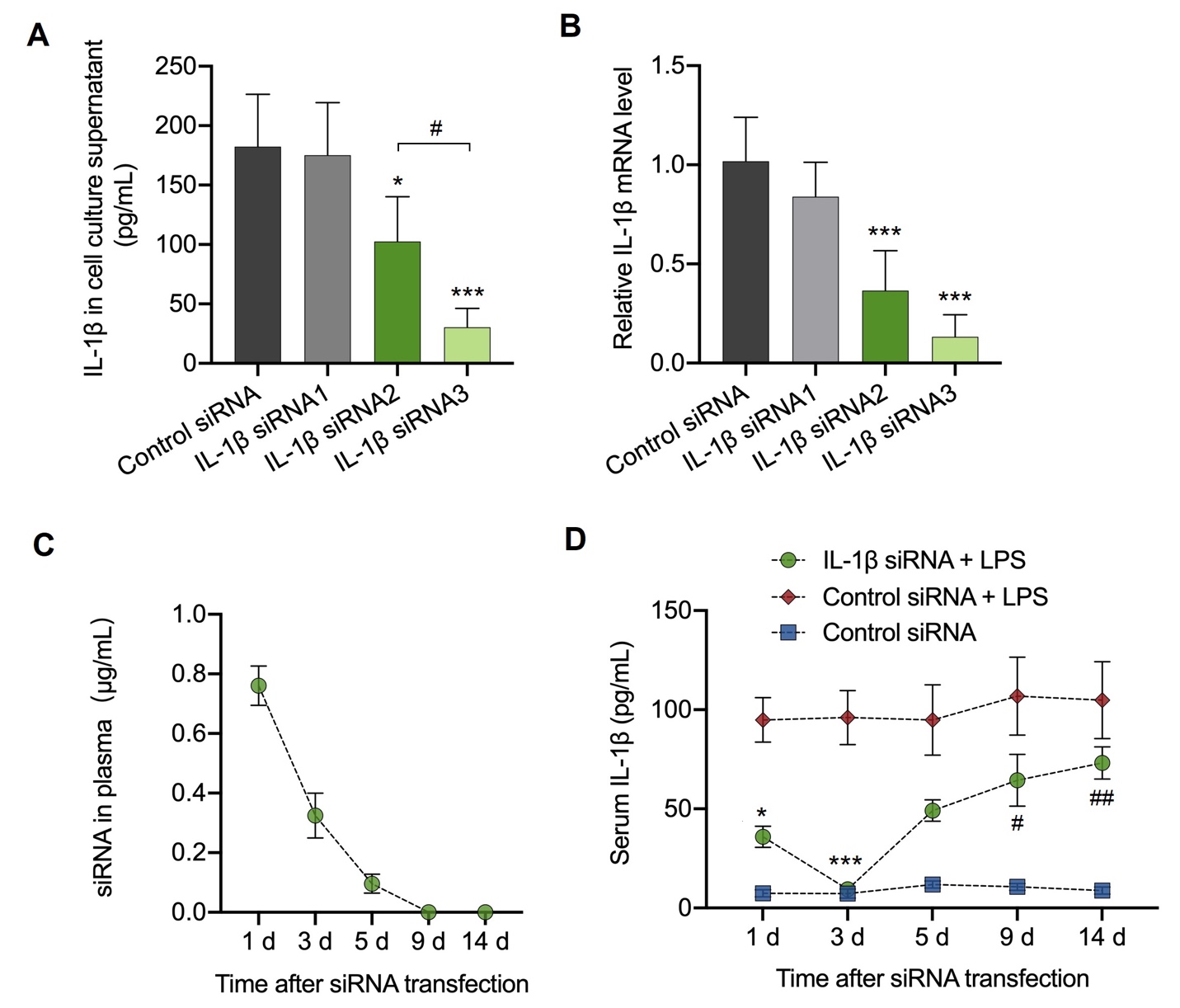
**

**Supplementary Figure 2:** Selecting and verifying of the chemically modified IL-1β siRNA sequence. (A. ELISA method to determine the IL-1β level in the supernatant of the mouse macrophage culture medium 48 h after siRNA transfection (LPS, Nigericin stimulation); B. Real-time PCR method to determine the relative expression of IL-1β RNA in mouse macrophage 48 h after siRNA transfection (LPS, Nigericin stimulation); C. The concentration curve of IL-1β siRNA in the plasma of mouse LPS model after tail vein injection; D. ELISA method to detect the knockdown effect of IL-1β siRNA in mouse LPS model after tail vein injection; *, P<0.05; **, P<0.01; ***, P<0.001.)

**
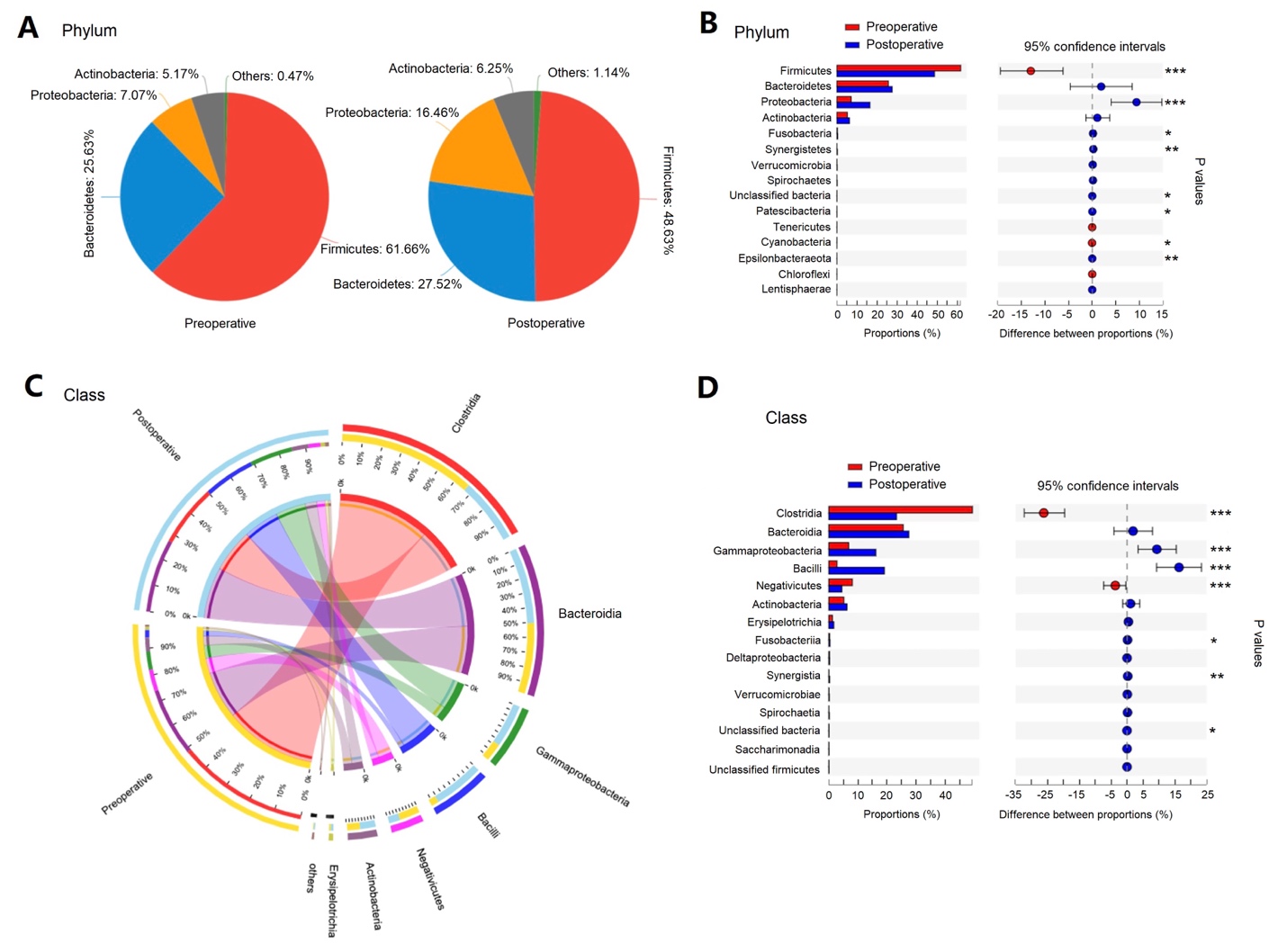
**

**Supplementary Figure 3:** Perioperative change of gut microbiota at the level of Phylum and Class. (A. Perioperative change in the composition of the patient’s gut microbiota at the phylum level; B. Forest plot of the perioperative change in the composition of the gut microbiota at the phylum level; C. Perioperative change in the composition of the patient’s gut microbiota at the class level; D. Forest plot of the perioperative changes in the composition of the gut microbiota at the class level; *: P < 0.05; **: P < 0.01; ***: P < 0.001)


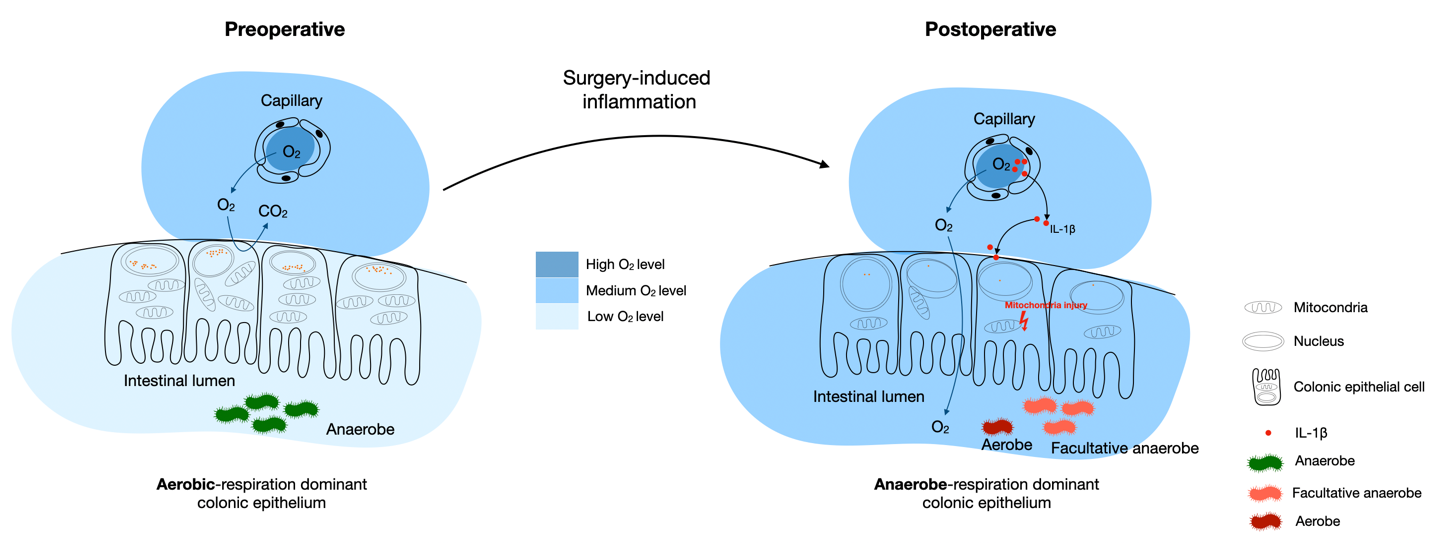


**Supplementary Figure 4:** The potentially underlying mechanism of gut dysbiosis after surgery/anesthesia stress (‘IL-1β - colonic epithelium oxygen metabolism - colonic oxygen environment – gut microbiota’ regulating axis). Left: During gut homeostasis, mature colonic epithelial cells primarily undergo aerobic respiration. The high oxygen consumption of colonic epithelial cells maintains the epithelial hypoxia (<1 % oxygen) to limit the amount of oxygen diffusing into the colon lumen, thus maintaining the homeostasis of the gut microbiota (predominant by anaerobes)[1]. Right: After surgery/anesthesia stress, high level of IL-1β induced mitochondrial damage in colonic epithelium and impairment of cellular aerobic respiration[2, 3]. As the oxygen consumption is reduced, excess oxygen diffuses into the colon lumen, destroying the hypoxic environment in colon lumen, which makes the colonic environment more livable for aerobic and facultative anaerobic bacteria and less livable for obligate anaerobic bacteria, thereby causing postoperative gut dysbiosis.


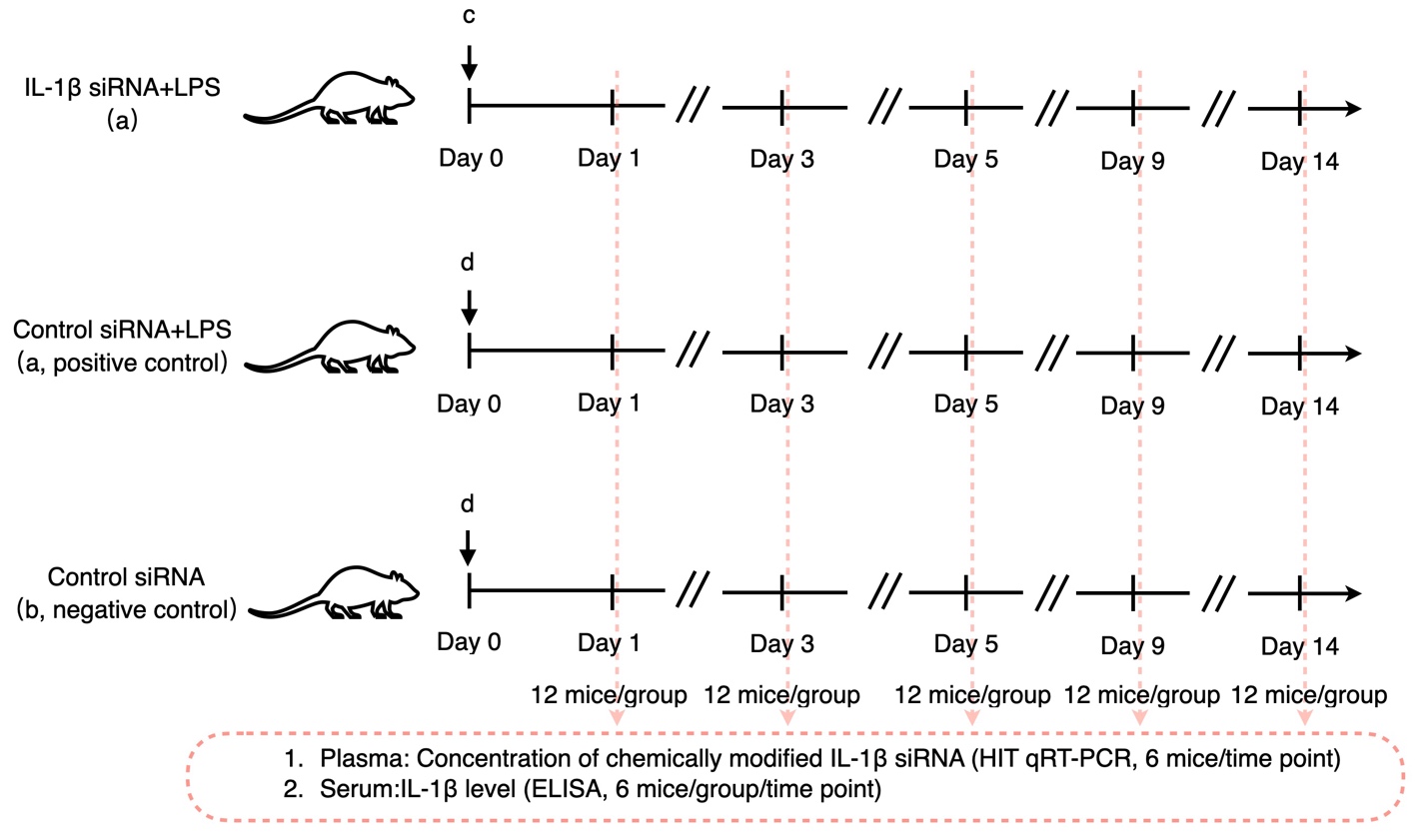


**Supplementary Figure 5:** *In vivo* knockdown effect validation of chemically modified IL-1β siRNA in a mouse LPS model. (a. Inject LPS intraperitoneal 6 h before euthanasia; b. Inject NS intraperitoneal 6 h before euthanasia; c. Tail vein injection of Invivofectamine™ 3.0/chemically modified IL-1β siRNA complexes; d. Tail vein injection of Invivofectamine™ 3.0/chemically modified control siRNA complexes.)

**Reference:**

1. Sheeran P, Hall GM. Cytokines in anaesthesia. British journal of anaesthesia. 1997;78(2):201-19.

2. Yasuhara R, Miyamoto Y, Akaike T, Akuta T, Nakamura M, Takami M, et al. Interleukin-1beta induces death in chondrocyte-like ATDC5 cells through mitochondrial dysfunction and energy depletion in a reactive nitrogen and oxygen species-dependent manner. Biochem J. 2005;389(Pt 2):315-23.

3. Kowluru RA, Mohammad G, Santos JM, Tewari S, Zhong Q. Interleukin-1β and mitochondria damage, and the development of diabetic retinopathy. J Ocul Biol Dis Infor. 2011;4(1-2):3-9.
